# Reproductive risk factors for endometrial cancer among Polish women

**DOI:** 10.1038/sj.bjc.6603731

**Published:** 2007-04-10

**Authors:** L A Brinton, L C Sakoda, J Lissowska, M E Sherman, N Chatterjee, B Peplonska, N Szeszenia-Dabrowska, W Zatonski, M Garcia-Closas

**Affiliations:** 1 Hormonal and Reproductive Epidemiology Branch, Division of Cancer Epidemiology and Genetics, National Cancer Institute, 6120 Executive Blvd., Suite 550, Rockville, MD 20852-7234, USA; 2 Department of Cancer Epidemiology and Prevention, Cancer Center and M. Sklodowska-Curie Institute of Oncology, W.K. Roentgena 5, Warsaw 02-781, Poland; 3 Biostatistics Branch, Division of Cancer Epidemiology and Genetics, National Cancer Institute, Rockville, MD 20852, USA; 4 Department of Occupational and Environmental Epidemiology, Nofer Institute of Occupational Medicine, Lodz, Poland

**Keywords:** endometrial cancer, reproduction, risk, abortion, breastfeeding

## Abstract

We conducted a population-based case–control study of reproductive factors in Warsaw and Ló∂ź, Poland, in 551 incident endometrial cancer cases and 1925 controls. The reproductive variable most strongly related to risk was multiparity, with subjects with three or more births having a 70% lower risk than the nulliparous women. The reduced risk was particularly strong below 55 years of age. Subjects with older ages at a first birth were also at reduced risk even after adjustment for number of births. Ages at last birth or intervals since last birth were not strongly related to risk. Spontaneous abortions were unrelated to risk, but induced abortions were associated with slight risk increases (odds ratios=1.28, 95% confidence intervals 0.8–2.1 for 3+ *vs* no abortions). The absence of effects on risk of later ages at, or short intervals since, a last birth fails to support the view that endometrial cancer is influenced by mechanical clearance of initiated cells. Alternative explanations for reproductive effects should be sought, including alterations in endogenous hormones.

Nulliparous women are known to be at an increased risk of endometrial cancer, but effects of other reproductive factors remain less defined. Inverse associations with later ages at a first birth have been demonstrated in some studies (Kvale *et al*, 1988; Albrektsen *et al*, 1995; Parslov *et al*, 2000; Wernli *et al*, 2006), although not in all of them (Lesko *et al*, 1991; Parazzini *et al*, 1991, 1998; Shu *et al*, 1991; Brinton *et al*, 1992; McPherson *et al*, 1996; Lambe *et al*, 1999). More recently, studies have suggested that risk may decrease with later ages at a last birth (Kvale *et al*, 1988, 1991; Lesko *et al*, 1991; Parazzini *et al*, 1991, 1998; Lambe *et al*, 1999). This may reflect the fact that late ages at a last birth tend to be associated with shorter intervals since last births (Albrektsen *et al*, 1995; Parazzini *et al*, 1998), prompting the suggestion that pre-malignant or initiated cells may be mechanically cleared during the birth process (Kvale *et al*, 1991; Lambe *et al*, 1999). Alternatively, women who are unable to give birth at later ages may be at high risk given anovulatory menstrual cycles and low associated progesterone levels (Kaaks *et al*, 2002).

Effects of other reproductive factors on endometrial cancer also remain unresolved. At least one study has suggested elevated risks among women with abortions late in reproductive life that are not followed by a subsequent term pregnancy (McPherson *et al*, 1996). Effects of breastfeeding on risk also remain controversial (Brinton *et al*, 1992; Rosenblatt and Thomas, 1995; Salazar-Martinez *et al*, 1999; Newcomb and Trentham-Dietz, 2000).

In many of the previous investigations, reproductive relationships may have been obscured by high rates of usage of exogenous hormones. In a recently completed study in Poland, where rates of exogenous hormone use are low, we had the opportunity to clarify a variety of unresolved reproductive issues related to endometrial carcinogenesis.

## MATERIALS AND METHODS

### Study subjects and data collection procedures

Eligible cases for this population-based case–control study consisted of residents of Warsaw and Ló∂ź, Poland, ages 20–74 years, with incident endometrial cancer diagnosed between June 1, 2001 and December 30, 2003. Cases were recruited through a rapid identification system organised at five participating hospitals, consisting of, in Warsaw, the Cancer Center, M. Curie Sklodowska Institute of Oncology Institute, and Polish Oncological Foundation, and in Lodz the Dr Madurowicz Memorial Hospital and Polish Mother's Health Memorial Hospital. These hospitals covered about 85% of all cases diagnosed in the two cities. Information from the Cancer Registries in Warsaw and Lodz was used to identify cases that were missed by the rapid identification system.

The Polish Electronic System (PESEL), a database with demographic information from all residents of Poland, was used to select controls. A complementary breast cancer study, initiated in January 2000, in which controls were randomly selected according to the anticipated age distribution (in 5 year categories) of breast cancer cases in each city provided a source for controls from the beginning of the endometrial cancer study until the end of the breast study (September 30, 2003). These controls were supplemented with additional subjects who were specifically selected for the endometrial cancer cases. One control per cancer case was selected during the breast cancer study, with two controls per case after this time. Eligible controls had neither a history of breast or endometrial cancer at enrolment.

After obtaining signed informed consent forms approved by the National Cancer Institute and local Institutional Review Boards (IRB), personal interviews were conducted using a questionnaire on demographic and reproductive factors, contraceptive behaviour, use of exogenous hormones, physical activity, passive and active cigarette smoking, occupational history, diet, alcohol use, first-degree family history of breast and ovarian cancer, medical and screening history, prenatal exposures and developmental history.

Of the 694 eligible cases and 2843 eligible controls, 551 (79.4%) and 1925 (67.7%), respectively, agreed to complete an interview. The primary reason for non-response was refusal, relevant to 106 (15.3%) of the eligible cases and 705 (24.8%) of the controls. The median length of the interview was 85 min, and the overall quality as rated by the interviewers was identical for the cases and controls, with 48.1% rated high quality, 49.1% generally reliable, 2.3% unreliable, and 0.5% unrated. The median time from case diagnosis to interview was 69.0 days and the median time from control selection to interview was 19.0 days. Medical records of endometrial cancer patients were reviewed for diagnostic and treatment details.

Information on all prior pregnancies was collected, including outcomes (livebirth, stillbirth, miscarriage, abortion, other), gestational lengths (in weeks or months), and dates each pregnancy ended. For livebirths, subjects were asked whether they had breastfed the baby and for how long. Subjects were also asked if they had ever tried to become pregnant for two straight years without successful conception and whether they had ever visited a doctor, clinic or hospital because of difficulty in becoming pregnant. If relevant, they were asked when they first sought advice and whether they were given any medication or hormones to help in getting pregnant.

### Statistical analysis

Unconditional logistic regression analysis was used to estimate adjusted odds ratios (OR) and associated 95% confidence intervals (CI). In order to examine the independent effects of correlated reproductive factors, we used the approach described by Heuch and others (Heuch *et al*, 1999), in which regression models were constructed using data from nulliparous and parous women.

## RESULTS

Information pertaining to the distribution of risk factors among the study participants is shown in Table 1. The mean age of the cases was 60.7 years, compared with 56.2 years among the controls. The majority of the study subjects derived from Warsaw. Cases tended to be somewhat better educated, more often menopausal, and to have earlier ages at menarche than controls. Although rates of usage were low, hormone replacement therapy (primarily combined oestrogen–progestin therapy) was more common among cases than controls, whereas the reverse was true regarding oral contraceptives. Cases were heavier than controls and were less often cigarette smokers, but there were no substantial differences between the two groups with respect to overall usage of alcoholic beverages.

A significantly decreased risk was associated with parity (OR_adj_ for parous *vs* nulliparous=0.51, 95% CI 0.4–0.7) (Table 2). In addition, risks decreased with increasing numbers of pregnancies (data not shown) and numbers of full-term births, with a slightly stronger relationship seen with the latter variable. The largest reduction in risk was for the initial birth (OR_adj_=0.60, 95% CI 0.4–0.8 for 1 birth *vs* 0 birth), although subsequent births were associated with continued reductions in risk (OR_adj_=0.30, 0.2–0.4 for 3 or more *vs* 0 births).

Among gravid women, a history of any spontaneous abortion was unrelated to risk, but there was a slightly increased risk for women who reported an induced abortion. However, there was no evidence that risk increased linearly with number of induced abortions, the risk for those with three or more induced abortions being similar to that with only one abortion (respective ORs of 1.28 (0.8–2.1) and 1.31 (1.0–1.7)). The risks associated with a history of induced abortion did not differ significantly by whether women were parous or nulliparous.

In contrast to the other reproductive parameters, a history of infertility (defined as the inability to get pregnant after trying to conceive for 2 years or longer) was only marginally related to risk and this association entirely disappeared after adjustment for other risk factors. A history of infertility was also not related to risk when defined more specifically according to whether physician advice had ever been sought or treatment received. Using various definitions, infertility was also not associated with risk when examined specifically among nulliparous women.

For consistency with most previous published analyses that have attempted to distinguish effects of correlated reproductive risk factors, further analyses focused on parous women (Table 3). In initial analyses, age at first birth did not appear related to risk, but after adjustment for other factors a trend of decreasing risk with increasing ages emerged. This showed that subjects with a first birth at 30 or later had an OR of 0.75 (95% CI 0.5–1.2) compared with those with a first birth before 20. Age at last birth appeared initially to be inversely related to risk, a trend that did not persist after adjustment for other factors. Interval since last birth was not related to endometrial cancer risk in any linear fashion before adjustment for other risk factors, but after adjustment for other factors (including parity, which was the major confounder), there was some evidence of increased risk with shorter intervals since last birth – with subjects whose last birth occurred within the last 20 years being at a nonsignificantly increased risk (OR=1.52, 95% CI 0.8–2.9) compared with those whose last birth occurred 40 or more years before diagnosis. When we further divided this group into those who had given birth within 15–20 years *vs* within 15 years, there were discrepant risks, being, respectively, 1.78 (0.9–3.4) *vs* 0.81 (0.3–2.0). However, this latter risk was based on only 10 cases and 220 controls. Among parous women, extended breastfeeding (24 or more months) appeared initially to be associated with a significant decrease in risk, but this relationship was attenuated after adjustment for a variety of other factors, including parity.

We also used a modelling approach (Heuch *et al*., 1999), which allowed us to simultaneously account for various time-related aspects of reproduction, including ages at first and last delivery (with this latter variable restricted to those with two or more births), and intervals since last birth (Table 4). Despite the different analytic approach, most results were similar to those derived from more traditional modelling approaches. Notably, parity persisted as the primary predictor of risk, with subjects with three or more births being at a 70% lower risk than nulliparous women (95% CI 0.2–0.5). Age at first birth also continued to show inverse associations with risk (OR=0.69 for subjects 30+ *vs* <20), whereas age at last birth was unrelated to risk. However, in contrast to our previous analyses, which suggested a possible increase in risk with years since last birth, these additional analyses showed no association with risk.

We also examined risks according to varying ages at diagnosis (<55, 55–65, 65+ years). As trends were very similar for the women 55–65 and those over 65, we combined these two groups for presentation (Table 5). The inverse relation with parity was strongest among the youngest women. Thus, having three or more full-term births was associated with a 93% reduction in risk among the subjects 55 years of age or younger, as compared with only a 57% reduced risk among the older subjects. However, among the older subjects, both parity and age at first birth were related to risk, with those having a first birth at 30 years of age or older having an OR of 0.65 compared with those with a first birth at 20 or younger. There were no striking relationships with age at last birth or time since last birth.

## DISCUSSION

Similar to previous investigations (Brinton *et al*, 1992; Albrektsen *et al*, 1995; Hinkula *et al*, 2002), we found a substantially reduced risk of endometrial cancer associated with parity, with women having three or more full-term births being at a 70% lower risk than nulliparous women. To shed further light on the effects of reproduction, we focused our analyses on the timing of births, which proved to be challenging, given the high degree of correlation of ages at and intervals since a last birth (Albrektsen *et al*, 1999). We used a modelling approach (Heuch *et al*, 1999) that enabled combining data from parous and nulliparous women to separately evaluate effects of different parameters.

We were particularly interested in following up the observations of an inverse relationship of endometrial cancer risk with ages at (Kvale *et al*, 1988; Lesko *et al*, 1991; Parazzini *et al*, 1991, 1998; Lambe *et al*, 1999) and intervals since (Albrektsen *et al*, 1995; Parazzini *et al*, 1998) a last birth. Our results, like others (Shu *et al*, 1991; McPherson *et al*, 1996; Albrektsen *et al*, 1999), provide little support for these relationships. Although we had few women with short intervals since a last birth, we saw no effects even among our youngest study subjects, in whom we had the greatest power to assess relations. Most studies that have observed relationships with intervals since a last birth have observed trends persisting over many years rather than being restricted to the postpartum period, raising questions about the biologic credibility of the hypothesis of mechanical clearance of precancerous cells during delivery.

It is important to note that several studies that have emphasised the importance of timing of the last birth have been unable to fully account for other predictors of endometrial cancer (Kvale *et al*, 1988; Albrektsen *et al*, 1995; Lambe *et al*, 1999). This includes oral contraceptives, a recognised protective factor for endometrial cancer, and an exposure that would undoubtedly be more prevalent among subjects with late ages at first birth. This was not an important confounder in our study, given low rates of usage among Polish women, but other studies that have adjusted for oral contraceptives have shown a persistent effect of ages at last birth (Lesko *et al*, 1991; Parazzini *et al*, 1998). It seems more likely that differing analytic techniques may explain study discrepancies, as an analysis that appropriately accounted for correlated variables also did not confirm an effect of late ages at a last birth (Albrektsen *et al*, 1999).

In contrast to the lack of association with ages at or intervals since a last birth, we observed that late ages at a *first* birth were associated with some risk reductions. This factor has not generally been regarded as a predictor of endometrial cancer risk (Pettersson *et al*, 1986; Lesko *et al*, 1991; Brinton *et al*, 1992; Parazzini *et al*, 1998; Xu *et al*, 2004), despite a number of studies that have demonstrated relatively strong associations (Kvale *et al*, 1988; Parslov *et al*, 2000; Hinkula *et al*, 2002; Wernli *et al*, 2006). Although it is widely accepted that multiparity may reduce endometrial cancer risk through changes in hormonal profiles, including lowered estradiol and increased sex hormone binding globulin levels (Chubak *et al*, 2004), underlying mechanisms for reduced risks associated with delayed ages at a first birth are less clear. This may relate to less frequent anovulation among women who can conceive at older ages (Escobedo *et al*, 1991; Modan *et al*, 1998). Future investigations should focus on hormonal changes associated with pregnancy, taking note of our findings as well as others (Pettersson *et al*, 1986; Albrektsen *et al*, 1995; Lambe *et al*, 1999) of stronger effects of multiparity among younger women.

The effects of infertility on endometrial cancer risk have been of considerable interest given that anovulatory menstrual cycles often reflect high exposure to oestrogens in the absence of sufficient progesterone. Elevated risks have been associated with delays in conception (Henderson *et al*, 1983; Shu *et al*, 1991), with one study showing a particularly high risk among nulliparous women (Brinton *et al*, 1992). Consistent with other investigations (Shu *et al*, 1991; McPherson *et al*, 1996), we found only a marginally elevated risk related to a physician diagnosis of infertility, with the relationship disappearing after adjustment for parity. The inconsistent findings across investigations may reflect varying definitions of infertility (Brinton *et al*, 2005). Given difficulties in obtaining accurate information from patients, future studies should focus on medically confirmed diagnoses, particularly those associated with hormonal alterations, such as polycystic ovarian disease, linked with uterine cancer risk elsewhere (Dahlgren *et al*, 1991; Escobedo *et al*, 1991; Modan *et al*, 1998; Pierpoint *et al*, 1998).

Our investigation also enabled evaluation of effects of short-term pregnancies, including spontaneous and induced abortions. Although several studies have noted either reduced (Parazzini *et al*, 1998; Parslov *et al*, 2000) or increased (Shu *et al*, 1991; McPherson *et al*, 1996) risks, we found no substantial relationship with spontaneous abortions, in line with most other investigations (Brinton *et al*, 1992; Xu *et al*, 2004; Wernli *et al*, 2006). Previous studies assessing the effects of induced abortion on endometrial cancer risk have been inconsistent, possibly reflecting small numbers of exposed women. The majority have shown either no effect (Parazzini *et al*, 1991; Brinton *et al*, 1992; Wernli *et al*, 2006) or possibly some reduction in risk (Shu *et al*, 1991; Parazzini *et al*, 1998; Parslov *et al*, 2000). We had considerable power to evaluate effects of induced abortions, given that they have been legal in Poland for many years. We observed a slightly increased risk associated with the reporting of an induced abortion, consistent with one previous study (McPherson *et al*, 1996). However, we observed no further risk increase among women with multiple abortions, raising questions regarding the biologic credibility of the relation. Reporting bias (as seen in breast cancer studies) (Rookus and van Leeuwen, 1996; Bartholomew and Grimes, 1998; Tang *et al*, 2000), uncontrolled confounding, or chance may be involved.

Several investigations have suggested that women with extensive breastfeeding histories may be at reduced endometrial cancer risk (Rosenblatt and Thomas, 1995; Salazar-Martinez *et al*, 1999; Newcomb and Trentham-Dietz, 2000; Wernli *et al*, 2006), possibly from suppression of ovarian hormones (Petrakis *et al*, 1987). Although we initially observed reduced risk among subjects who breastfed for 2 or more years, this relation disappeared after adjustment for other risk factors. However, as in most studies in developing countries, we had few women who breastfed for extended periods of time.

In sum, this study confirmed that endometrial cancer is strongly related to parity. Using analytic techniques which overcome previous difficulties in assessing correlated reproductive variables, we found that late ages at a first birth also appeared to reduce risk, but that late ages at a last birth or extended intervals since a last birth were unrelated. Endometrial cancer risk did not appear to be substantially influenced by incomplete pregnancies, histories of infertility, or years of breastfeeding. Future investigations should focus on hormonal alterations underlying multiparity and late ages at a first birth in order to clarify processes involved in endometrial carcinogenesis.

## Figures and Tables

**Table 1:** Distribution of non-reproductive risk factors among study participants

	**Cases (*n*=551)**		**Controls (*n*=1925)**	
**Study variables**	**Number**	**Percent**	**Number**	**Percent**
*Age, years*				
<55	133	24.1	889	46.2
55–64	206	37.4	547	28.4
65+	212	38.5	489	25.4
				
*Study site*				
Warsaw	393	71.3	1316	68.4
Lodz	158	28.7	609	31.6
				
*Years of education*				
<High school	188	34.1	727	37.8
High school	207	37.6	728	37.8
Some college or professional training	152	27.6	460	23.9
				
*Age at menarche, years*				
<13	155	28.1	412	21.4
13	138	25.0	428	22.2
14	135	24.5	546	28.4
15+	120	21.8	513	26.7
				
*Regular menstrual cycles*				
Yes	470	85.3	1648	85.6
No	81	14.7	277	14.4
				
*Menopause status*				
Premenopausal	93	16.9	623	32.4
Postmenopausal	416	75.5	1199	62.3
				
*Use of oral contraceptives*				
No	520	94.4	1703	88.5
Yes	28	5.1	199	10.3
				
*Use of hormone replacement therapy*				
No	404	73.3	1469	76.3
Yes	140	25.4	431	22.4
				
*Cigarette smoking*				
Non-smoker	358	65.0	898	46.6
Past	92	16.7	356	18.5
Current	101	18.3	671	34.9
				
*Alcohol intake, drinks/week*				
None	412	74.8	1295	67.3
<2	72	13.1	296	15.4
2–<3	19	3.4	88	4.6
3–<4	12	2.2	51	2.6
⩾4	18	3.3	140	7.3
				
*Recent body mass index, kg m* ^−*2*^				
<23.05	81	14.7	461	23.9
23.05–25.94	120	21.8	476	24.7
25.95–29.17	136	24.7	473	24.6
29.18+	208	37.7	469	24.4

Missings excluded from presentation, but included in denominator for calculation of above percentages. Numbers of missing for cases *vs* controls were as follows: age (0,0), years of education (4,10), age at menarche (3,26), menopausal status (7,112), use of oral contraceptives (3,23), use of hormone replacement therapy (7,25), cigarette smoking (0,0), alcohol intake (18,55), recent body mass index (6,46).

**Table 2:** Relationships of reproductive factors to endometrial cancer risk

**Risk factors**	**No. of cases (*n*=551)**	**No. of controls (*n*=1925)**	**Age and site-adjusted OR (95% CI)**	**Fully adjusted OR (95% CI)^a^**
*Ever had a full-term birth*				
No	102	218	1.00	1.00
Yes	449	1707	0.53 (0.4–0.7)	0.51 (0.4–0.7)
				
*Number of full-term births*				
0	102	218	1.00	1.00
1	171	544	0.63 (0.5–0.8)	0.60 (0.4–0.8)
2	225	850	0.54 (0.4–0.7)	0.52 (0.4–0.7)
3+	53	313	0.31 (0.2–0.5)	0.30 (0.2–0.4)
			*P*(*trend)*<*0.0001*	*P*(*trend)*<*0.0001*
				
*Number of spontaneous abortions* b				
0	367	1348	1.00	1.00
1	81	327	0.88 (0.7–1.2)	0.84 (0.6–1.1)
2+	34	98	1.23 (0.8–1.9)	1.10 (0.7–1.7)
			*P*(*trend)*=*0.82*	*P*(*trend)*=*0.72*
				
*Number of induced abortions* b				
0	301	1259	1.00	1.00
1	93	275	1.34 (1.0–1.8)	1.31 (1.0–1.7)
2	58	164	1.32 (0.9–1.8)	1.31 (0.9–1.9)
3+	30	75	1.33 (0.8–2.1)	1.28 (0.8–2.1)
			*P*(*trend)*=*0.02*	*P*(*trend)*=*0.05*
				
*Ever problem with infertility*				
No	498	1769	1.00	1.00
Yes	52	150	1.33 (0.9–1.9)	1.03 (0.7–1.5)
Unknown	1	6		

a Fully adjusted model includes age, site, years of education, age at menarche, number of full-term births, ever use of oral contraceptives, ever use of oral hormones, ever smoking, recent body mass index.

b Restricted to gravid women.

**Table 3:** Relationships of additional reproductive factors to endometrial cancer risk among parous women

**Risk factors**	**No. of cases (*n*=449)**	**No. of controls (*n*=1707)**	**Age and site-adjusted OR (95% CI)**	**Fully adjusted OR (95% CI)^a^**
*Age at first birth*				
<20	64	226	1.00	1.00
20–24	238	864	1.02 (0.7–1.4)	0.93 (0.7–1.3)
25–29	104	450	0.88 (0.6–1.3)	0.74 (0.5–1.1)
30+	43	167	1.00 (0.6–1.6)	0.75 (0.5–1.2)
			*P*(*trend)*=*0.59*	*P*(*trend)*=*0.08*
				
*Age at last birth*				
<25	154	495	1.00	1.00
25–29	160	625	0.88 (0.7–1.1)	0.95 (0.7–1.2)
30–34	93	381	0.85 (0.6–1.1)	1.01 (0.7–1.4)
35+	42	206	0.70 (0.5–1.0)	0.87 (0.6–1.3)
			*P*(*trend)*=*0.06*	*P*(*trend)*=*0.67*
				
*Years since last birth*				
40+	145	327	1.00	1.00
30–39	142	436	0.86 (0.6–1.2)	1.02 (0.7–1.4)
20–29	117	496	1.12 (0.7–1.7)	1.55 (0.9–2.5)
<20	45	448	0.92 (0.5–1.6)	1.52 (0.8–2.9)
			*P*(*trend)*=*0.98*	*P*(*trend)*=*0.13*
				
*Months of breastfeeding*				
None	83	319	1.00	1.00
<12	218	903	0.87 (0.6–1.2)	0.90 (0.7–1.2)
12–23	113	308	1.12 (0.8–1.6)	1.35 (0.9–1.9)
24+	35	177	0.57 (0.4–0.9)	0.72 (0.4–1.2)
			*P(trend)*=*0.24*	*P(trend)*=*0.88*

a Fully adjusted model includes age, study, site, years of education, age at menarche, number of full-term births, ever use of oral contraceptives, ever use of oral hormones, ever smoking, recent body mass index.

**Table 4:** Multivariate model of risk related to various correlated reproductive factors

**Risk factors**	**No. of cases (*n*=551)**	**No. of controls (*n*=1925)**	**Age and site-adjusted OR (95% CI)**	**Fully adjusted OR (95% CI)^a^**
*Number of full-term births*				
0	102	218	1.00	1.00
1	171	544	0.84 (0.5–1.4)	0.78 (0.5–1.3)
2	225	850	0.59 (0.4–0.9)	0.54 (0.3–0.9)
3+	53	313	0.33 (0.2–0.6)	0.30 (0.2–0.5)
				
*Age at first birth* b				
<20	64	226	1.00	1.00
20–24	238	864	0.88 (0.6–1.2)	0.87 (0.6–1.2)
25–29	104	450	0.69 (0.5–1.0)	0.67 (0.4–1.0)
30+	43	167	0.78 (0.5–1.3)	0.69 (0.4–1.2)
				
*Age at last birth* c				
<25	59	230	1.00	1.00
25–29	115	452	1.25 (0.8–1.8)	1.22 (0.8–1.8)
30–34	73	309	1.34 (0.8–2.1)	1.36 (0.8–2.2)
35+	31	172	1.13 (0.6–2.0)	1.06 (0.6–1.9)
				
*Years since last birth* b				
40+	145	327	1.00	1.00
30–39	142	436	0.84 (0.6–1.2)	0.91 (0.6–1.3)
20–29	117	496	1.00 (0.6–1.6)	1.10 (0.7–1.8)
<20	45	448	0.79 (0.4–1.5)	0.96 (0.5–1.9)

a Fully adjusted model includes age, study site, years of education, age at menarche, ever use of oral contraceptives, ever use of oral hormones, ever smoking, recent body mass index.

b For parous women.

c For women with more than one full-term birth.

**Table 5:** Relationships of reproductive factors to endometrial cancer risk among parous women by varying ages at diagnosis

	**Study subjects <55 years of age (133 cases, 889 controls)**		**Study subjects 55+ years of age (418 cases, 1036 controls)**	
	**No. cases, no. controls**	**OR^a^ (95% CI)**	**No. cases, no. controls**	**OR^a^ (95% CI)**
*Number of full-term births*				
0	39, 99	1.00 (referent)	63, 119	1.00 (referent)
1	36, 252	0.31 (0.1–0.8)	135, 292	1.10 (0.6–1.9)
2	51, 411	0.17 (0.05–0.5)	174, 439	0.77 (0.4–1.3)
3+	7, 127	0.07 (0.02–0.3)	46, 186	0.43 (0.2–0.8)
				
*Age at first birth*				
<20	8, 93	1.00 (referent)	56, 133	1.00 (referent)
20–24	49, 404	1.19 (0.5–2.8)	189, 460	0.82 (0.5–1.2)
25–29	25, 210	0.91 (0.3–2.4)	79, 240	0.62 (0.4–1.0)
30+	12, 83	0.94 (0.3–3.0)	31, 84	0.65 (0.3–1.2)
				
*Age at last birth* b				
<25	6, 92	1.00 (referent)	53, 138	1.00 (referent)
25–29	27, 224	1.76 (0.6–4.7)	88, 228	1.17 (0.7–1.8)
30–34	18, 143	2.34 (0.7–7.4)	55, 166	1.26 (0.7–2.1)
35+	7, 79	1.52 (0.4–5.9)	24, 93	1.17 (0.6–2.3)
				
*Years since last birth*				
25+	28, 170	1.00 (referent)	334, 832	1.00 (referent)
20–24	26, 201	1.01 (0.5–2.0)	16, 56	0.96 (0.5–1.9)
<20	40, 419	1.17 (0.5–2.8)	5, 29	0.59 (0.2–1.8)

a Fully adjusted model includes age, study site, years of education, age at menarche, ever use of oral contraceptives, ever use of oral hormones, ever smoking, recent body mass index.

b For women with more than one full-term birth.
